# Factors affecting the cost-effectiveness of on-farm culture prior to the treatment of clinical mastitis in dairy cows

**DOI:** 10.1016/j.prevetmed.2017.07.006

**Published:** 2017-09-15

**Authors:** P.M. Down, A.J. Bradley, J.E. Breen, M.J. Green

**Affiliations:** a University of Nottingham, School of Veterinary Science and Medicine, Sutton Bonington Campus, Sutton Bonington, Loughborough LE12 5RD, United Kingdom; b Quality Milk Management Services Ltd, Cedar Barn, Easton Hill, Easton, Wells BA5 1DU, United Kingdom

**Keywords:** Mastitis, On-farm culture, Cost-effectiveness, Treatment, Probabilistic sensitivity analysis

## Abstract

The objective of this study was to use probabilistic sensitivity analysis to evaluate the cost-effectiveness of using an on-farm culture (OFC) approach to the treatment of clinical mastitis in dairy cows and compare this to a ‘standard’ treatment approach. A specific aim was to identify the herd circumstances under which an OFC approach would be most likely to be cost-effective. A stochastic Monte Carlo model was developed to simulate 5000 cases of clinical mastitis at the cow level and to calculate the associated costs simultaneously when treated according to 2 different treatment protocols; i) a 'conventional' approach (3 tubes of intramammary antibiotic) and ii) an OFC programme, whereby cows are treated according to the results of OFC. Model parameters were taken from recent peer reviewed literature on the use of OFC prior to treatment of clinical mastitis. Spearman rank correlation coefficients were used to evaluate the relationships between model input values and the estimated difference in cost between the standard and OFC treatment protocols. The simulation analyses revealed that both the difference in the bacteriological cure rate due to a delay in treatment when using OFC and the proportion of Gram-positive cases that occur on a dairy unit would have a fundamental impact on whether OFC would be cost-effective. The results of this study illustrated that an OFC approach for the treatment of clinical mastitis would probably not be cost-effective in many circumstances, in particular, not those in which Gram-positive pathogens were responsible for more than 20% of all clinical cases. The results highlight an ethical dilemma surrounding reduced use of antimicrobials for clinical mastitis since it may be associated with financial losses and poorer cow welfare in many instances.

## 1 Introduction

Mastitis is one of the most prevalent and costly diseases affecting dairy cows worldwide. The cost of clinical mastitis is made up of ‘direct’ costs, such as the cost of drugs, discarded milk and increased labour and ‘indirect’ costs, such as reduced future production, increased culling and increased the risk of disease transmission to herd mates. The overall cost of a case of clinical mastitis has been shown to be highly variable (Huijps et al., 2008, Green et al., 2009) and most influenced by ‘indirect’ costs (Kossaibati and Esslemont, 2000, Huijps et al., 2008, Down et al., 2013).

Not only is mastitis important in terms of the economics, but the treatment and prevention of mastitis is widely reported as the most common reason for antimicrobial drug use on dairy farms (Pol and Ruegg, 2007, Thomson et al., 2008, González et al., 2010). There is increasing pressure on the agricultural sector to reduce antimicrobial drug usage due to fears over antimicrobial resistance (AMR) (O’Neill, 2015) and the way in which antimicrobial drugs are applied with respect to the treatment of mastitis is, therefore, a sensible target. Conventionally, all cases of clinical mastitis would receive a course of antimicrobial agents but an alternative approach is the selective treatment of cases according to the results of an on-farm culture (OFC) system. With the OFC system, only non-severe cases that yield a Gram-positive or mixed-culture are treated with antimicrobial drugs resulting in many cases of clinical mastitis not being treated at all (Lago et al., 2011a). This was demonstrated recently in a study performed in 8 herds based in Minnesota, Wisconsin and Ontario, which reported that 51% of cows enrolled in the OFC group received antimicrobial drugs as opposed to 100% of the cows enrolled in the conventional group. The same study reported no statistical differences between the two groups with respect to the bacteriological cure risk, the time taken to clinical cure, new intramammary infection risk, treatment failure risk or risk of removal from the herd within 21 days (Lago et al., 2011a).

While OFC appears to be effective in reducing antimicrobial drug usage; little is known about factors influencing the overall cost-effectiveness of this approach and, therefore, the types of herds in which it is most likely to be cost-effective. When performing such a cost-effectiveness analysis, there are inevitably multiple sources of evidence for parameter estimates and a degree of uncertainty surrounding their true value (Ades et al., 2006). For decision-making purposes, it is important that cost-effectiveness models are able to incorporate multiple sources of evidence and reflect uncertainty in the model outputs (Claxton et al., 2005, Babo Martins and Rushton, 2014). An approach to this now widely reported in the human health literature (Briggs et al., 2002, Brown et al., 2006) and increasingly in the veterinary literature (Down et al., 2013, Hudson et al., 2014, Hudson et al., 2015) is probabilistic sensitivity analysis (PSA). The main feature of this technique is that all input parameters are specified as full probability distributions, rather than point estimates, to represent the uncertainty surrounding their values. This parameter uncertainty is then propagated through the cost-effectiveness model so that the resulting imprecision is reflected in model outputs and inferences made (Briggs et al., 2002).

The purpose of this research was to use probabilistic sensitivity analysis to investigate the main factors that influence the cost-effectiveness of an OFC approach to treating clinical mastitis. The model used was an adaptation of one reported previously (Down et al., 2013), with the addition of OFC-specific parameters based on previous research (Lago et al., 2011a). A specific aim was to identify the herd circumstances under which an OFC approach would be most likely to be cost-effective.

## 2 Materials and methods

### 2.1 Model structure

A stochastic Monte Carlo model was developed using OpenBUGS 3.2.2 software (Thomas et al., 2006). The model was used to simulate a case of clinical mastitis at the cow level and to calculate the associated costs simultaneously when treated according to 2 different treatment protocols; i) a ‘conventional’ approach (3 tubes of intramammary antibiotic) and ii) an OFC programme as described by Lago et al. (2011a) in which milk samples taken from cows with CM were cultured on-farm using the Minnesota Easy Culture System (University of Minnesota, St. Paul). This on-farm milk culture system consisted of a bi-plate, which is a Petri dish with two different types of agar: MacConkey agar on one half that selectively grows Gram-negative bacteria and Factor media on the other half of the plate that selectively grows Gram-positive bacteria. The plate was placed in an on-farm incubator and incubated at approximately 37 °C for 18–24 h. The next day, the plate was examined for bacterial growth and interpreted by herd personnel and if bacteria did not grow, the plate was returned to the incubator and re-read approximately 18–24 h later. Results for each sample plate were recorded as (1) Gram-positive, when bacteria grew only in the Factor agar media of the bi-plate; (2) Gram-negative, when bacteria grew only in the MacConkey agar media of the bi-plate; (3) no growth, when bacteria did not grow on either media; or (4) mixed infection when bacteria grew on both media. Quarters from which Gram-positive bacteria were isolated or had a mixed infection received intramammary antibiotic treatment and if the on-farm milk culture result was Gram-negative or no growth, the quarter did not receive intramammary therapy.

**Fig. 1:**
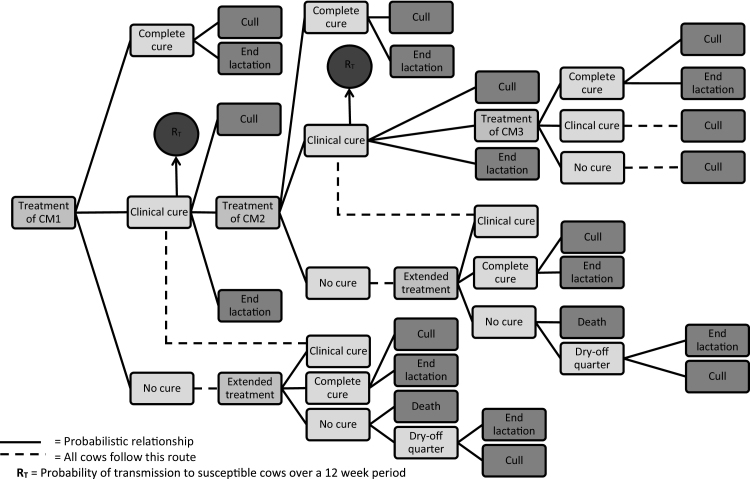
Schematic representation of the treatment model. Complete cure = bacteriological and clinical cure. Clinical cure = non-bacteriological cure but clinical cure. No cure = no bacteriological or clinical cure. Cull = culled sometime within the remainder of the current lactation. Extended treatment = a repeat of the same treatment that the case received initially. R_T_ = risk of transmission. CM1 = initial case of clinical mastitis. CM2 = first clinical flare-up. CM3 = secon clinical flare-up.

The general model structure and assumptions were consistent irrespective of the treatment protocol applied and was as described previously by Down et al. (2013) (Fig. 1). An initial case of clinical mastitis (CM1) could either; i) cure bacteriologically, ii) cure clinically but remain subclinically infected, or iii) fail to cure (either clinically or bacteriologically). If CM1 failed to cure, then a repeat treatment (same as initial treatment) would be administered and the same three outcomes permitted. If CM1 cured bacteriologically, then the cow could either end the lactation or be culled before the end of lactation. If CM1 cured clinically but not bacteriologically, then it could either end the lactation, be culled before the end of lactation, or have a repeat episode of clinical mastitis (CM2). CM2 would be treated according to the same protocol as CM1 and would then follow the same possible outcomes as CM1. A third clinical recurrence was permitted for subclinically infected cows (CM3) which were again treated in the same way as CM1 and CM2. If the cow remained subclinically infected after CM3 or failed to cure clinically, then the cow would be culled before the end of lactation. If the cow cured bacteriologically after CM3, then it could either finish the lactation or be culled before the end of lactation.

A risk of transmission parameter was included from cows that remained subclinically infected after CM1 and CM2. This represented the risk that the infection was transmitted from the infected cow to one of the other 99 ‘susceptible’ cows in the herd during a 12-week period. The 12-week period was split into 14-day blocks meaning the infected cow could infect another cow in the herd every 14-days. If infection did spread to another cow, then it too would be considered to be infectious during the subsequent 14-day blocks. For example, if a cow remained subclinically infected after CM1, and it transmitted an infection to another cow during the first 14-day block, then there would be two infectious cows at the start of the second 14-day block, and the susceptible population would be 98 cows. However, it should be noted that the transmission parameter would be applicable to herds of different sizes and the results of the model could be extrapolated to herds of any size.

### 2.2 Model input parameters

The model was parameterized with distributions based on existing literature and current commercial data where possible (Table 1). All parameter inputs were specified as uniform distributions with the purpose of simulating a wide variety of different scenarios without making assumptions as to which was the most likely. The distribution ranges were based on the literature wherever possible, but if only point estimates were available, then plausible ranges were added to the point estimate. The input parameters were the same as used in the study by Down et al. (2013), with the addition of some OFC-specific parameters based on the study by Lago et al. (2011a).

**Table 1:** Probability distributions applicable to both treatment protocols and relevant sources of literature on which they are based where applicable.

Input parameters	Upper and lower limits of uniform distribution	Source
Probability of bacteriological cure (0.40,0.80)		a, b, c, d, e, f, g, h
Probability of bacteriological cure after extended tx (0.30,0.90)		Based upon Steeneveld et al. (2011)
Decrease in probability of bacteriological cure^1^		Based upon Steeneveld et al. (2011)
Parity ≥2	(−0.15,−0.05)	
Days in milk ≥60 days	(−0.15,−0.05)	
Cow is systemically ill	(−0.25,−0.15)	
SCC 200,000–500,000 cells/mL at most recent recording	(−0.15,−0.05)	
SCC >500,000 cells/mL at most recent recording	(−0.25,−0.15)	
Repeated case (>1st case in current lactation)	(−0.25,−0.15)	
Probability of being culled for bacteriologically noncured cases		Based upon Bar et al. (2008)
Initial case	(0,0.32)	
Following first flare-up (CM2)	(0.04,0.36)	
Probability of being culled for completely cured cases		Based upon Bar et al. (2008)
Initial case	(0.04,0.06)	
Following first flare-up (CM2)	(0.10,0.20)	
Following second flare-up (CM3)	(0.20,0.30)	
Probability of death for nonclinical cured cases	(0.04,0.06)	Based upon Bar et al. (2008)
Probability of drying-off quarter for nonclinical cured cases	(0.94,0.96)	Based upon Steeneveld et al. (2011)
Probability of being culled for cows with dried off quarters	(0.27,0.39)	Based upon Steeneveld et al. (2011)
Increase in all culling probabilities when cow is systemically ill	(0.05,0.15)	Based upon Steeneveld et al. (2011)
Probability of clinical flare-up for bacteriologically noncured cases	(0.05,0.12)	m, n, o
Probability of transmission after CM1 and CM2	(0.002,0.25)	van den Borne et al. (2010)
Proportional yield loss		j, k
Case in 1st or 2nd month of lactation	(0.07,0.09)	j, k, l
Case between months 3 and 6	(0.03,0.08)	
Case after month 6	(0,0.04)	
Parity ≥2	(0,0.02)	
305d Yield (Kg)	(7000,10,000)	Author^2^
Milk withdrawal (d)	(5.00,9.00)	Based upon commonly used preparations in the UK
Daily milk discard (Kg)	(5.00,50,00)	Author^2^
Value of discarded milk ($/Kg)	(0.33,0.39)	DairyCo (2012a)
Cost of milk production ($/Kg)	(0.043,0.145)	Based upon Huijps et al. (2008)
Treatment Time (hr)	(0.53,0.87)	Based upon Steeneveld et al. (2011)
Cost of labour ($/hr)	(1.45,23.01)	Based upon Huijps et al. (2008)
Cost of drugs ($)	(8.10,10.11)	Based upon estimate of current retail price of commonly used preparations in the UK
Cost of cull ($)	(174,1044)	Based upon Huijps et al. (2008), Kossaibati and Esslemont (2000)
Cost of death ($)	(1740,2900)	DairyCo (2012b)

a Based upon McDougall (1998), b Based upon McDougall (2003), c Based upon Oliver et al. (2003), d Based upon Wraight (2003), e Based upon Sérieys et al. (2005), f Based upon McDougall et al. (2007), g Based upon Bradley and Green (2009), h Based upon Sol et al. (2000), j Based upon Gröhn et al. (2004), kBased upon Schukken et al. (2009), l Based upon Hagnestam et al. (2007), m Based upon Swinkels et al. (2005a), n Based upon Swinkels et al. (2005b), o Based upon Döpfer et al. (1999).

1 The value selected from this distribution was subtracted from the value selected from the bacteriological cure distribution.

2 Author – where there was no relevant literature identified on which to base the parameter, distributions were based on biologically plausible values instead.

Economic parameter distributions included the cost of drugs, labour, milk withdrawal and loss of milk production, culling and death (Table 1). The cost of labour is subject to large variation quoted in the literature. For this reason a wide distribution was assigned to the hourly cost of labour with the upper limit taken from Huijps et al. (2008). The total time taken to treat each case of CM was assigned a distribution centred on the figures given by Steeneveld et al. (2011) surrounded by an additional variation of +/− 10 minutes. The total cost of labour was the product of the hourly rate and the total treatment time.

The length of milk withdrawal after CM was defined by a distribution based on the commonly used medicines in the UK and the amount of milk being discarded each day was taken from a plausible milk yield distribution (Table 1). The distribution defined for milk price was taken from DairyCo (2012a) and based on the average UK milk price over the last 12 months (range: lowest price and highest price). The cost of milk production was based on Huijps et al. (2008) and assigned a uniform distribution to reflect the variability in the figure (Table 2).

**Table 2:** On-farm culture specific model input parameters and the relevant sources of literature on which they were based where applicable.

Input parameters	Upper and lower limits of uniform distribution	Source
Proportion of Gram-positive cases	(0.10,0.90)	Based upon Lago et al. (2011a)
Reduction in bacteriological cure risk	(−0.22,0.00)	Based upon Lago et al. (2011a)
Cost of plate ($)	(1.45,2.03)	Based upon current retail price
Culture time (h)	(0.30.1.00)	Expert opinion

The calculation of total yield loss following a case of CM was based on the herd 305 day yield, the parity of the animal and the stage of lactation in which the infection occurred (Table 1). The distributions governing the percentage of total loss in 305 day milk yield were based on Hagnestam et al. (2007). The proportion of cases occurring at each stage postpartum and the proportion of cases affecting multiparous cows versus primiparous cows was governed by distributions based on Steeneveld et al. (2011) (Table 1). The cost associated with the total loss in milk yield was calculated according to the total loss in earnings (i.e. the quantity of milk multiplied by the milk price) minus the savings made in feed costs (i.e. the quantity of milk loss multiplied by the cost of production). All distributions are provided in Table 1.

The cost of culling a cow within the remainder of the current lactation was taken from a uniform distribution based on Huijps et al. (2008) and Kossaibati and Esslemont (2000), which included the slaughter value and replacement costs, with an appropriate range added to reflect the variability of this parameter (Table 1). The cost of the death of an individual was based on current UK average sales prices for freshly calved cows and heifers (DairyCo, 2012b) which would be required to replace the dead cow in addition to the cost of carcass disposal (Table 1).

The original calculations were made in Great British Pounds (**£**) and converted to US Dollars (**$**) using the exchange rate of 1.45 $/£ (http://www.xe.com/currencyconverter/; accessed 15th February 2016).

### 2.3 On-farm culture specific input parameters

The OFC-specific input parameters comprised distributions reflecting changes to the bacteriological cure risk, the proportion of culture-positive cases, the time taken to set-up and read the culture plates and the cost of a plate. The distribution for the reduction in bacteriological cure risk associated with the OFC protocol was uniform (−0.22 to 0), meaning the maximum reduction possible was 22%, and the minimum was 0. The middle value of 11% was the non-significant effect size reported by Lago et al. (2011a) which was the overall reduction in bacteriological cure risk in cases of clinical mastitis treated with the OFC protocol compared to cases treated with the conventional approach. The distribution specifying the ‘herd-level’ proportion of Gram-positive cases was uniform (0.1–0.9), meaning the lowest proportion was 10% of cases with a Gram-positive culture and the highest proportion was 90%. This distribution reflects the wide spread of values identified in the study by Lago et al. (2011a). There were no published figures for the cost of the biplate used in the study or the time taken to set-up and evaluate the culture results so plausible ranges were estimated as ($1.45–2.03) and (30–60 min) respectively. The distributions used for all other input parameters are listed in Table 1.

### 2.4 Model simulation

The model was used to simulate 5000 cases of CM1 for each treatment protocol which was sufficient for all combinations of treatment scenarios and other input parameters to be effectively investigated so that dependencies could be evaluated. At each model-iteration, a value was selected at random from within the ranges specified for each input parameter, independent of each other, and the associated costs calculated. The parameter values and overall cost were stored for each model-iteration and were used for subsequent analysis. The outcome of interest was the difference in overall cost between the two protocols which was calculated at each model-iteration by subtracting the cost of the OFC approach from the cost of the conventional approach. Therefore, a positive value would indicate that the conventional approach was more cost-effective and a negative value would indicate that the OFC protocol was more cost-effective. The distribution specifying the herd-level proportion of Gram-positive cases would govern whether the case treated according to the OFC protocol was Gram-positive (or mixed infection) and, therefore, treated with antibiotics or Gram-negative (or no growth) and, therefore, not treated with antibiotics. In this way, the impact of the proportion of Gram-positive cases on the overall cost-effectiveness of the OFC protocol could be assessed.

### 2.5 Data analysis

Spearman rank correlation coefficients were calculated to explore the univariate associations between model input parameters and the difference in cost between the conventional and OFC treatment protocols. The strength and direction of the relationships were evaluated using the Spearman rank rho (ρ) value. The outcome variable of specific interest was the difference in cost between the two treatment protocols. However, additional model parameters were included to provide further insight into where cost differences arose. These were the cost of antimicrobial drugs, the difference in time taken to treat each case, the difference in milk withdrawal period and the difference in the risk of transmission.

Descriptive analysis was performed to identify scenarios in which the OFC approach was most cost-effective. To facilitate this, the 5000 simulated cases were sub-divided into three groups according to the magnitude of reduction in bacteriological cure risk associated with the OFC protocol as compared with the conventional approach; i) large difference (LD) group (17–22% reduction), ii) moderate difference (MD) group (5–17% reduction) and iii) small difference (SD) group (0–5% reduction). The difference in cost-effectiveness between the conventional and OFC protocols was then assessed for the different groups and at different proportions of Gram-positive cases.

## 3 Results

### 3.1 Data analysis

Across all 5000 simulated cases, the conventional protocol was the most cost-effective 68% of the time. The median cost related to a case treated with the conventional protocol was $529, and the median cost related to a case treated with the OFC protocol was $554. The maximum difference in cost between the two protocols was $328 with a median of $28.

The Spearman rank correlation coefficients for the OFC-specific parameters are shown in Table 3. The difference in cost between the two protocols was most closely related to the difference in bacteriological cure risk and the proportion of Gram-positive cases. As the difference in bacteriological cure risk and proportion of Gram-positive cases increased, the difference in overall cost became higher, making the OFC protocol less cost-effective than the conventional protocol. Both the cost of the biplate and the time taken to set-up and evaluate the biplate had a negligible relationship with the cost-effectiveness of the OFC protocol as measured by the Spearman rank correlation coefficients (Table 3).

**Table 3:** Spearman rank correlation coefficients for on-farm specific model input parameters and the difference in cost between the conventional and on-farm culture treatment protocols.

Parameter	rho
Proportion culture-positive	0.31
Difference in bacteriological cure risk	−0.28
Cost of plate	0.0062
Culture time	0.02

With respect to the model input parameters common to both protocols, those significantly associated with the difference in cost were the difference in the milk withdrawal period (rho = 0.75), difference in the cost of drugs (rho = 0.61), difference in the time taken to treat the cow (and culture) (rho = 0.61) and the difference in the risk of transmission (rho = 0.51).

### 3.2 Scenario and sensitivity analysis

**Fig. 2:**
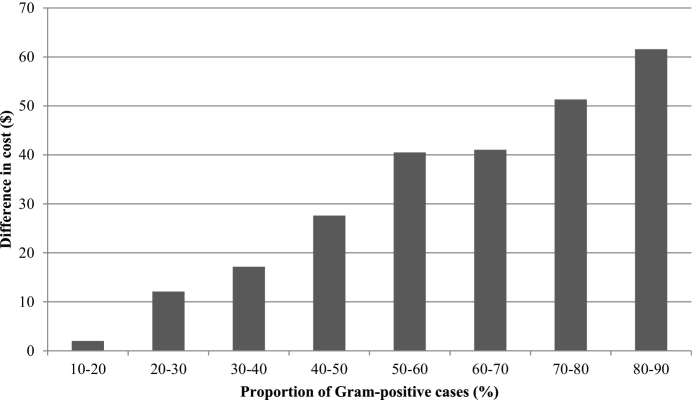
Difference in cost between conventional and on-farm culture protocols. A positive difference in cost indicates that the on-farm culture protocol cost more than the conventional protocol.

**Fig. 3:**
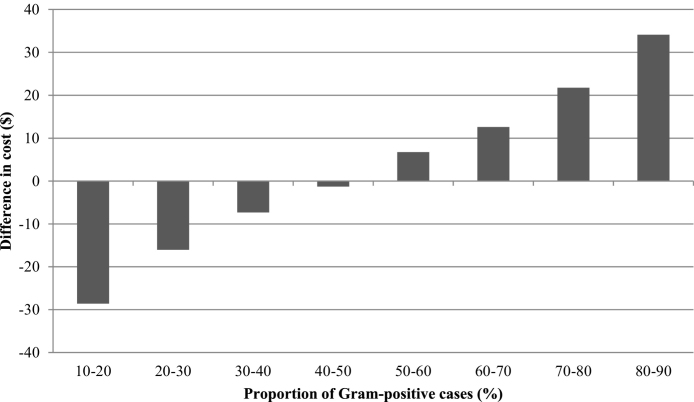
Difference in cost between conventional and on-farm culture protocols when there was a small difference (SD) in bacteriological cure risk. A positive difference in cost indicates that the on-farm culture protocol cost more than the conventional protocol.

**Fig. 4:**
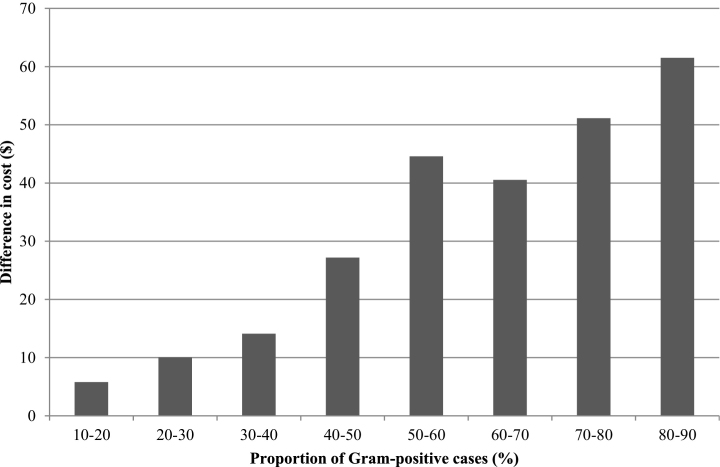
Difference in cost between conventional and on-farm culture protocols when there was a moderate difference (MD) in bacteriological cure risk. A positive difference in cost indicates that the on-farm culture protocol cost more than the conventional protocol.

**Fig. 5:**
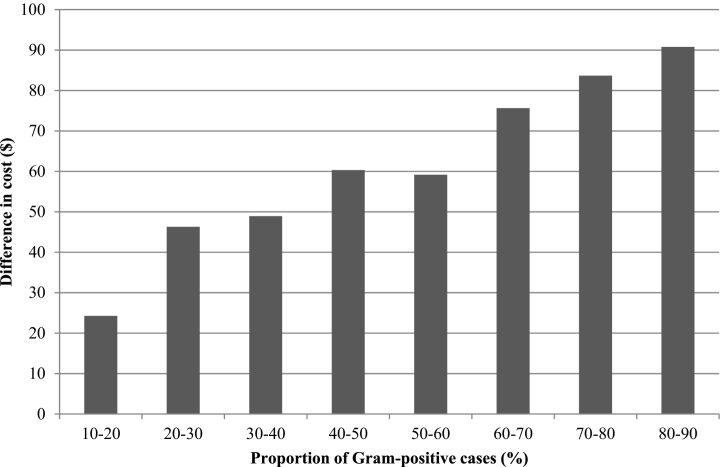
Difference in cost between conventional and on-farm culture protocols when there was a large difference (LD) in bacteriological cure risk. A positive difference in cost indicates that the on-farm culture protocol cost more than the conventional protocol.

The median difference in cost between the two protocols was plotted against the proportion of Gram-positive cases, and this indicated that the proportion of Gram-positive cases would need to be less than 12% for the OFC protocol to be more cost-effective than the conventional protocol (Fig. 2). When the proportion of Gram-positive cases increased to 50%, the OFC protocol was on average $42 more expensive per case than the conventional protocol. However, the difference in cost between the treatment groups was sensitive to the underlying bacteriological cure risk of Gram-positive cases. When clinical mastitis was subdivided according to whether the difference in bacteriological cure risk was small (SD) medium (MD) or large (LD) the difference in the cost-effectiveness of the treatments was as follows. The OFC protocol was more cost-effective than the conventional protocol when the proportion of Gram-positive cases was less than 47% in the SD group (Fig. 3) and less than 21% in the MD group (Fig. 4). The OFC protocol was never more cost-effective than the conventional protocol for cases in the LD group (Fig. 5). Therefore, the underlying bacteriological cure risk was a key parameter determining relative cost-effectiveness of the treatment approaches.

## 4 Discussion

The simulation analyses revealed that both the difference in the bacteriological cure risk due to a delay in treatment and the proportion of Gram-positive cases that occur on a dairy unit will have a fundamental impact on whether OFC will be cost-effective. There has undoubtedly been a shift in the aetiology of clinical mastitis towards environmental pathogens, with coliforms and no-growths frequently reported as accounting for approximately 50% of all clinical mastitis culture results (Bradley and Green, 2001, Bradley et al., 2007, Breen et al., 2009) as was the case in the study by Lago et al. (2011a). On this basis, it would be fair to assume that most dairy herds would expect to treat approximately 50% of clinical mastitis cases with antimicrobial drugs if utilizing an OFC approach. The reduction in bacteriological cure risk associated with OFC is more difficult to predict as there is very little published data on the extent to which cure is reduced when basing treatment decisions on the results of OFC. However, a reduction of some degree is possible given the 24 h delay in initiating antimicrobial treatment for the Gram-positive cases and the potential for Gram-positive cases to be incorrectly diagnosed as Gram-negative and therefore not treated, as was the case in 14% of the cases not treated with antibiotics in the study by Lago et al. (2011a). Given the results of this research, further work to quantify any likely reduction in bacteriological cure risk that is associated with OFC is critical, if the cost-effectiveness and welfare implications of OFC are to be established.

One of the aims stated by the authors of the original OFC studies (Lago et al., 2011a, Lago et al., 2011b) was to use their results to evaluate the overall cost-benefit of using an OFC system, but to date, no data have been published. In a decision tree analysis used to evaluate the economic impact of different durations of intramammary treatment for the first case of mild or moderate clinical mastitis occurring in early lactation, it was found that OFC was only likely to offer economic benefits in herds using extended-duration therapy without regard for pathogen diagnosis (Pinzón-Sánchez et al., 2011). The results of this study serve to illustrate that an OFC approach for the treatment of clinical mastitis would probably not be cost-effective in many circumstances, in particular, not those in which Gram-positive pathogens represent more than 20% of all clinical cases. Since *Streptococcus uberis* and *Staphylococcus aureus* remain common mastitis pathogens on dairy units in many countries, the cost-effectiveness of OFC should be carefully scrutinised in these circumstances.

While OFC will reduce total antimicrobial drug usage on farm, the effect on cow health and welfare and overall dairy farm profitability should also be considered. The assertion that there is no important reduction in bacteriological cure from delayed treatment of Gram-positive pathogens is fragile and requires substantially more research with sufficient power to detect small differences in effect size. In the study by Lago et al. (2011a), statistical analysis revealed a non-significant difference of 11% in bacteriological cure risk between the conventional and OFC groups. In that study, the sample size used meant that a difference ≥14% would have been needed between groups to detect the difference as being ‘significant’ (Lago et al., 2011a) and it, therefore, remains uncertain whether there is a true difference in bacteriological cure between groups. The sensitivity analysis in the current study suggests that a difference in cure risk of less than 14% could certainly determine whether OFC is cost-effective and, therefore, larger studies to ascertain this true difference are needed.

Significant differences were reported in the pathogen-specific bacteriological cure risk in the study by Lago et al. (2011a), particularly with respect to *Staphylococcus aureus*. While the reason for these differences is unknown, it is possible that the reduction in bacteriological cure risk associated with OFC is a result of delayed treatment as was hypothesised by Lago et al. (2011a) and has been reported in a previous study (Hillerton and Semmens, 1999). Given the importance of this parameter, future research should include pathogen-specific differences in bacteriological cure risk, when treatment is delayed by using OFC.

In the current study, the overall proportion of Gram-positive cases was also shown to be related to the likelihood of cost-effectiveness of an OFC treatment programme. The proportion of Gram-positive cases was shown to be highly variable in the study by Lago et al. (2011a) in which the proportion of quarter cases receiving intramammary antibiotic treatment as a consequence of assignment to the OFC protocol ranged from 31%–89% in the eight study herds. In the current study, the overall proportion of Gram-positive cases had to be less than 12% (depending on bacteriological cure risk) for OFC to be more cost-effective than the conventional protocol. By this measure, OFC would not have been cost-effective in any of the herds in the study by Lago et al. (2011a). However, when cases were grouped according to the difference in bacteriological cure risk, OFC would be cost-effective when the proportion of Gram-positive cases was less than 47% in the SD group and less than 21% in the MD group. The OFC approach would, therefore, be most suitable for herds in which Gram-negative pathogens are responsible for most clinical mastitis and where the treatment of cows using an OFC approach results in a minimal reduction in the bacteriological cure risk. In practice, it is possible to assess the proportion of Gram-positive cases on a unit, and this will inform decision making on the likely cost-benefit of OFC.

There may be a balance to be struck between reducing antimicrobial usage and possible deleterious effects in terms of cow welfare and farm finances; would the extra cost incurred by adopting an OFC approach be considered a price worth paying if it results in a reduction in antibiotic drug usage on dairy farms by 25%, as was estimated by Lago et al. (2011a)? If, for societal reasons, this was considered to be a price worth paying, there is also an issue of who should bear the cost. Whilst difficult, it is perhaps time such debates became transparent given the increasing pressure on antimicrobial drug usage and the potential risks posed by antimicrobial resistant bacteria. In the absence of legal jurisdiction, it is incumbent on those advising on animal health and welfare to ensure that the adoption of new technologies, such as OFC, are undertaken in light of comprehensive, transparent welfare and cost-effectiveness assessments.

Whilst the overall likelihood of cost-effectiveness was affected mostly by the proportion of Gram-positive cases and the difference in bacteriological cure risk, the parameters within the model that had the largest impact on the difference in cost were the difference in milk-withdrawal period, the difference in the cost of drugs, the difference in culture and treatment time and the difference in risk of transmission. Clearly, OFC would be expected to reduce the amount of milk withdrawn from sale and the amount of money spent on drugs because a proportion of the cows would not receive any antimicrobial treatment and would therefore not incur any statutory milk withhold upon resolution of clinical signs. This is in agreement with Lago et al. (2011a) that reported a reduction in milk withdrawal period (5.2 days v 5.9 days) and quantity of antimicrobial drug usage (51% of OFC cases treated v 100% of conventional cases treated) associated with OFC. The increase in labour required to acquire milk samples from clinical mastitis cases in an aseptic manner and plate out for culture is perhaps harder to assess and is likely to represent a cost not only in terms of the time taken, but also the opportunity cost incurred as a result of the herdsman being unable to perform other duties as a result. The distribution used in this study of 30–60 min is, therefore, likely to be a realistic estimate for most circumstances. The large impact that transmission could have on the cost of a case of clinical mastitis has been reported previously (Down et al., 2013) and it is not surprising therefore that it was closely related to the difference in cost between the conventional and OFC approaches also. While the risk would clearly be influenced by herd management and pathogen-specific factors; it could also be affected by any delay in treatment and differences in bacteriological cure risk associated with OFC resulting in an increased risk of transmission. This again would need to be assessed at the herd level. It is worth noting that milk price had very little impact on the difference in cost between the two protocols. This is unsurprising given the poor correlation between milk price and the cost of a case of CM that has been reported previously (Down et al., 2013) and serves to demonstrate that whilst important in terms of overall farm profitability (Smith and Thanassoulis, 2015), milk price plays a minor role in terms of the cost of CM.

There will inevitably be some unknown parameters in any cost-effectiveness model (Buxton et al., 1997) and these parameters will have a degree of uncertainty surrounding their true value. PSA permits the incorporation of this parameter uncertainty which is subsequently propagated through the model and is therefore reflected in the model outputs. It is widely considered to be an implementation of Bayesian statistics because all parameters have a probability distribution which is a distinguishing feature of the Bayesian approach (O’Hagan, 2003, Boshuizen and van Baal, 2009). One of the key advantages of the Bayesian approach in medicine is that it removes the reliance on significance testing and the use of arbitrary thresholds of ‘significance’ (Gurrin et al., 2000, Greenland and Poole, 2013) meaning the clinician is free to make their own judgement as to what is clinically ‘significant’ according to the degree of uncertainty they are comfortable with. In this study, the PSA allowed an evaluation of the parameters likely to be important in determining the cost-effectiveness of the OFC approach and has highlighted that more research is needed in this field before the technique can be recommended on a widespread basis.

## 5 Conclusions

The results of this study indicate that the proportion of Gram-positive cases and the difference in bacteriological cure risk between the two treatment approaches has the greatest impact on the probability that an OFC approach would be more cost-effective than a conventional approach for the treatment of clinical mastitis. The OFC approach appears to be suitable for herds in which Gram-negative pathogens are responsible for most clinical mastitis and where the treatment of cows according to the results of an OFC approach results in minimal reductions in the bacteriological cure risk. These results suggest that OFC will probably not be cost-effective for many herds and that OFC should, therefore, only be adopted after careful consideration of the predominant pathogens present in each herd and an honest discussion about the uncertainty surrounding its overall cost-effectiveness.

## Conflict of interest statement

The authors have no conflicts of interest
